# GERIATRICS PREVENTABLE ADMISSIONS CARE TEAM (GERIPACT): A HIGH-RISK INTENSIVE AMBULATORY GERIATRICS PROGRAM

**DOI:** 10.1093/geroni/igz038.529

**Published:** 2019-11-08

**Authors:** Stephanie W Chow, Lizette Munoz, Susana Lavayen, Shamsi Fani, Blair MacKenzie, Audrey Chun

**Affiliations:** 1 Icahn School of Medicine at Mount Sinai, New york, New York, United States; 2 Mount Sinai Hospital, New York, New York, United States

## Abstract

The Geriatrics Preventable Admissions Care Team (GERIPACT) is an inter-professional team of 2 clinicians, 1 social worker, and 1 care coordinator, dedicated to offering temporary intensive ambulatory care services to complex older patients at high-risk for incurring expensive health care (ie. frequent emergency room visits or hospitalizations). GERIPACT services include frequent office visits for medical and social work needs, frequent telephone contact, home visits, specialty visit accompaniment, and a 24/7 telephone hotline. Use of this innovative model aims to serve communities lacking in geriatrician and geriatric social work providers, with a main goal of serving the highest risk older population. We reviewed the healthcare utilization of GERIPACT enrollees 6 months prior-to-enrollment and compared with 6 months following graduation from GERIPACT from 2016 to 2018. 78 patients were evaluated, with 49 total ED visits prior to enrollment and 35 post-graduation, saving 14 ED visits for a ratio of 18 saved ED visits per 100 GERIPACT patients. There were 45 hospitalizations prior to enrollment with 29 hospitalizations post-graduation, saving 16 hospitalizations, or 20 hospitalizations per 100 GERIPACT patients. Hospital days were reduced by 237 days post-graduation. An intensive ambulatory program for high risk geriatrics patients may be shown to be an efficient model of care for targeting those older patients who potentially incur greater expenses to the health care system. This focused team may be deployed to primary care communities with complex elderly patients in need of geriatricians and geriatric social workers, and may reduce unnecessary emergency room visits and inpatient stays.

